# Antibiotic prophylaxis prior to urodynamic study in patients with traumatic spinal cord injury. Is there an indication?

**DOI:** 10.1590/S1677-5538.IBJU.2018.0574

**Published:** 2019-04-01

**Authors:** Marcello Torres da Silva, André Luis Barboza, Maria Malen Pijoán, Paulo Sergio Siebra Beraldo

**Affiliations:** 1 Serviço de Urologia, Rede Sarah de Hospitais de Reabilitação, São Luís, MA, Brasil;; 2 Serviço de Urologia, Rede Sarah de Hospitais de Reabilitação, Brasília, DF, Brasil;; 3 Instituto Universitario Italiano de Rosario - Ciências Biomédicas, Rosario, Santa Fe, Argentina;; 4 Serviço de Lesão Medular, Rede Sarah de Hospitais de Reabilitação, Brasília, DF, Brasil

**Keywords:** Urinary Tract Infections, prevention and control [Subheading], Spinal Cord Injuries

## Abstract

**Study design:**

Retrospective cohort of patients with traumatic spinal cord injury (SCI) that have been hospitalized for physical-functional rehabilitation purposes.

**Objectives:**

To compare the incidence of urinary tract infection (UTI) after urodynamic study (UDS) in three hospitals that adopted different protocols with regard to the preparation of patients.

**Setting:**

Sarah Network of Rehabilitation Hospitals, Brazil.

**Materials and Methods:**

Between 2014 and 2015, 661 patients from three units of the same hospital network, one of which does not use antimicrobial prophylaxis independently of urine culture results, were evaluated after having undergone UDS. The results were compared in both univariate and multivariate analyses (logistic regression).

**Results:**

The global rate of UTI after UDS was that of 3.18% (IC 95% 2.1-4.8), with no differences between the units. In the univariate analysis the only variable that was associated with UTI after UDS was that of T6 injuries or above (P = 0.029). The logistic regression has confirmed this result, with an adjusted odds ratio of 3.06 (IC 95% 1.01 to 9.26; P = 0.0476). The use of antimicrobial prophylaxis did not alter that risk.

**Conclusions:**

This study has demonstrated that the use of antimicrobials does not prevent UTI after UDS. Patients with T6 traumatic SCI or above have got three times more chance of developing UTI after UDS if compared to those with a T7 injury or below, independently of the use of antimicrobials. Even in these patients the use of antimicrobials would not be justified.

## INTRODUCTION

The traumatic spinal cord injury (SCI) generates a high socio-economic impact, since it usually focuses on individuals in their productive phases, who start to demand constant medical attention for the rest of their lives (1). Independently of the level of the injury, these patients very often present neurogenic bladder dysfunction, with the necessity of frequent urologic exams, notedly urodynamic study (UDS). This exam is safe and has a low potential of causing bacteremia. On the other hand, these patients frequently empty their bladders with the aid of a clean intermittent catheterization (CIC), which usually leads them to develop asymptomatic bacteriuria (1).

Bacterial resistance to antimicrobials is a public health problem worldwide. Its main cause is the indiscriminate prescription of antimicrobials, which calls our attention to the importance of a constant review in prescription policies (2, 3). Patients with SCI are more susceptible to recurrent urinary tract infection (UTI) and / or vesical colonization by multiresistant germs (4).

Although a sole clinical trial suggests the use of prophylactic antimicrobials prior to UDS (5), there are still controversies with regard to the indication or not of its routine usage by such patients. Since we count on the fact that we have conducted UDS in patients with SCI from three units of a same hospital network, each following its own protocols, the efficacy of such protocols was analyzed before the UDS. We have considered the incidence of UTI as the primary outcome, and, as independent variables, apart from the participant hospital units, characteristics related with the individual and with the SCI.

## MATERIALS AND METHODS

Between January, 2014, and December, 2015, three units of the Sarah Network of Rehabilitation Hospitals, Brazil, which adopted different routine protocols before UDS, were considered. The protocols were authorized by each unit's respective hospital-acquired infection committees. The delineation of this investigation is, thus, typical of a retrospective cohort, with no interference with the patient's preparation routine. The investigation project was submitted to the Ethics Committee of the Sarah Network, which approved it with no restrictions.

Six hundred and sixty one subsequent patients with traumatic SCI were analyzed, 197 (29.8%) of which came from the São Luis unit (henceforth called Hospital A), 328 (49.6%) from the Salvador unit (B) and 136 (20.6%) from the Brasilia unit (C). All the patients were hospitalized and participated in the rehabilitation program, which had an average duration of 30 to 45 days. All the data were collected, retrospectively, based on the information obtained from the electronic medical records and evaluation protocols of each respective hospital. We have considered as independent variables, apart from the unit where the UDS was conducted, the result of the basal urine culture, the sex and age of the patient, the level and age of the injury, the American Spinal Injury Association classification (ASIA), the presence of spasticity and the order of the exam (first or second). None of the patients has taken part more than once in the analysis.

Prior to the UDS, information concerning all the patient's urine and urine culture were collected. Patients with skin lesions, UTI and indwelling catheterization were excluded from the analysis, as well as outpatients, due to the difficulty in clinical observation. A culture was considered positive whenever any kind of bacterial growth was detected. In addition, the development of symptoms up to five days after the UDS, along with a new positive urine culture, were considered as UTI. Amongst the symptoms of UTI, we have considered malodor, macroscopic alterations, increases in urinary incontinence, dysuria (in patients with incomplete injuries), increases in spasticity, aggravations in the neuropathic pain, malaise, autonomic dysreflexia or fever with no other apparent cause (6).

In short, the protocols of the units involved consisted of:

– Hospital A: patients with a positive urine culture are submitted to antibiotic prophylaxis based on the urine culture, in a single dose, one hour before the UDS, while those with a negative urine culture receive 100 mg of nitrofurantoin.
– Hospital B: none of the patients is submitted to antibiotic prophylaxis before the UDS, independently of the result of the urine culture (positive or negative).
– Hospital C: patients with a positive urine culture are submitted to antibiotic prophylaxis based on the urine culture, in a single dose, two hours before the UDS, while those with a negative urine culture do not get prophylactic antibiotics.

In all of the three units, the UDS was carried out by professionals trained inside the Sarah Network, according to the ICS standards (7).

The data collected were compiled in the application Microsoft Excel. For the statistical analysis of proportions we used the chi-squared distribution study, while for the analysis of continuous variables we applied the Kruskal-Wallis test. The cutting criterion to consider a variable in the multivariate analysis was that of P < 0.10. The binary logistic regression analysis was carried out through the stepwise method (forward selection) with the aid of the application SPSS (version 17). The logistic model has allowed for an estimate of the probability of occurrence of UTI after the UDS, based on the presence of risk factors included in the analysis. The evaluation for the adjustment or adaptation of the model was obtained through the Hosmer-Lemeshow method (8). The probability of a type I error to occur was considered that of P < 0.05.

## RESULTS

The global UTI rate observed after the UDS was that of 3.18% (CI 95% 2.1-4.8), without distinctions between Hospitals A (2.53%), B (3.35%) or C (3.67%).

In the univariate analysis, considering the outcome of interest (presence or absence of UTI against variables such as sex, level of the injury (dichotomized as paraplegia or tetraplegia), motor injury completeness (complete or incomplete), presence of spasticity, use of antimicrobials prior to the study (Hospitals A and C), method of bladder emptying (spontaneous voiding x CIC), positivity or negativity of the basal urine culture and order of the UDS (first or second exam), no association was pointed out (Table-1).

**Table 1:** Categorical variables in the groups of patients with and without urinary tract infection after the UDS, with their respective odds ratios (OR; univariate analysis), confidence interval of 95% (CI 95%) and statistical significance.

Variables	Category	Urinary Tract Infection		OR	CI 95%	P value
		Yes	No			
**Sex**						
	Female	2	110			
	Male	19	530	1.97	0.45-8.59	0.36
**Level of the injury**						
	Tetraplegia	9	188			
	Paraplegia	12	452	0.56	0.23-1.34	0.19
**Motor injury** *						
	Complete (AIS A/B)	19	504			
	Incomplete (AIS C/D/E)	1	129	4.86	0.65-36.67	0.13
**Spasticity**						
	Flaccid	2	101			
	Present	17	436	1.99	0.45-8.66	0.37
**Use of antimicrobials**						
	No	13	363			
	Yes	8	277	0.81	0.33-1.97	0.64
**Bladder emptying**						
voluntary urination		5	170			
intermittent urinary catheterization		16	470	1.16	0.42-3.21	0.78
Basal urine culture						
	Negative	5	120			
	Positive	16	520	0.74	0.27-2.06	0.56
**Order of the UDS**						
	Second	13	371			
	First	8	269	0.85	0.35-2.08	0.72

* International Standards for Neurological and Functional Classification of SCI. (Maynard, 1997 286 /id)

Some of these and other variables were reviewed with regard to the existence of a possible relationship with UTI appearing after the UDS. Most of the injuries were caused by fire guns (203 cases, 30.7%), motor vehicle collisions (301 cases, 45.5%), falling from high heights (112 cases, 16.9%) and trauma over the dorsal region (40 cases, 6.1%), none of them being associated with the outcome of interest.

The patient's average age was that of 35.7 ± 11.1 years, with no distinctions between the hospitals. The patient's ages were divided in three groups for the UDS to be carried out: up to 30 years of age (237 patients, 50.1%), between 30 and 50 years of age (344, 50%) and above 50 years of age (80, 12.1%). No relationship between the patient's ages and the development of UTI was detected. The patients had been injured for an average time of 37.7 months (interquartile range 17.5 – 96.9). This average was lower amongst patients from Hospital C (P < 0.001). In any way, when we stratified the time since injury in up to two years, (222 patients, 33.6%), between 2.1 and 5 years (190, 28.7%) and above 5 years (249, 37.6%), we have also not found any associations with the outcome of interest.

Specifically with regard to the level of the injury, there were 116 patients (17.6%) with high cervical SCI (C1-C5), 73 (11.0%) with low cervical SCI (C6-C8), 193 (29.2%) with high thoracic SCI (T1-T6) e 279 patients (42.2%) with injuries beneath the T6 level. In no one of those four categories we observed any associations between the development of UTI and the UDS. On the other hand, dichotomizing the level of the injury between T6 or above (382 patients, 57.8%) and T7 or below (279, 42.2%), we have found that the first group presented a greater chance of developing UTI after UDS (4.5% against 1.4%, P = 0.029).

As for the completeness of the SCI, based on the ASIA Impairment Scale (AIS), we have classified 441 (66.7%) patients as AIS A, 82 (12.4%) as AIS B, 83 (12.5%) as AIS C and 47 (7.1%) as AIS D. In eight of the cases it was not possible to reach a classification. Once more, no relationship was found with the incidence of UTI after UDS.

After adjustments concerning the confounders from Table-1, which were carried out due to the UDS and included the patient's age and time since injury, both in years, we have not identified any independent variables. Similarly, when we tested, instead of the usage of prophylactic antibiotics, the hospital where the exam was carried out, no independent variables were identified. The only simulation that showed an association between the development of UTI and the UDS occurred when we substituted in the model the level of injuries, paraplegia or tetraplegia, by injury above or below the T6 level, which gave us an adjusted odds ratio of 3.06 (CI 95% 1.01 to 9.26; P = 0.0476). That is, independently of the usage of antimicrobials or of the hospital where the UDS was conducted, patients with a T6 level injury or above have got three times the chance of developing UTI after the exam, in relation to those with a T7 level injury or below.

## DISCUSSION

This observational multicenter research trial, involving 661 patients with traumatic SCI from three units of the Sarah Network of Rehabilitation Hospitals, has not demonstrated the necessity of antimicrobials in the prevention of UTI as a complication of UDS. The only independent variable associated with the outcome was the level of the SCI: T6 or above.

Patients with neurogenic bladder due to SCI frequently present asymptomatic bacteriuria, with no formal indication in their treatments (9). We know that this colonization can even exert a protective effect against the growth of other pathogenic agents, having proposals of inoculating low-virulence bacteria in neurogenic bladders already been made (10).

About three quarters of the studied patients used CIC as their main aid of bladder emptying and eight out of ten of those patients presented asymptomatic bacteriuria. These patients recur to CIC at least five times a day with single use catheter without the usage of prophylactic antibiotics. Both the chosen method of bladder emptying and asymptomatic bacteriuria were not independent risk factors for the development of UTI in this study, results which have already been shown by other authors (11).

The only clinical trial conducted more than two decades ago with the specific aim of answering this question has counted on just forty patients and concluded that the prophylactic antimicrobial should be indicated (5). From then on innumerable other observational research studies were carried out in order to confirm such results, since many researchers have questioned the possibility of a type I error, due to the diminished size of the sample or to the presence of potential confounders. Up to nowadays the literature is scarce with regard to the usage of prophylactic antimicrobials prior to UDS in these patients (12). The American Society of Urology recommends prophylaxis in a patient with neurogenic bladder when uroculture is positive which in fact occurs in the vast majority of cases (13).

Uncertainties involving the best conduct in the usage of antimicrobials before UDS have lead to the adoption of different conducts between the three units of the same network of rehabilitation hospitals in which we work. This situation has created the natural opportunity to compare the results obtained by each of them, with an expressive number of patients, and, with the pertinent statistical adjustments, to allow for valid conclusions, even with a retrospective observational design. All three units admit patients with traumatic SCI and neurogenic bladder, with similar clinical and neurological profiles. The only exception was the smaller amount of time since injury amongst patients from Hospital C. The three hospitals also carry the exams out by a team that was trained within the Sarah Network itself, with the same technical rigor. The main difference between the three units is the protocol for the patient's antibiotic prophylaxis before the UDS. Hospital B does not use any antimicrobials on its patients, even when they present asymptomatic bacteriuria, while the other two hospitals do use antimicrobials one to two hours before, depending on the result of the basal urine culture. Even with such peculiarities, we have not found differences on the incidence of UTI, either when we analyzed the three hospitals or when we dichotomized them into the two units that make usage of antimicrobials (Hospitals A and C) and the unit that does not use them (Hospital B).

We have found a global rate of UTI after UDS of 3.18%, which is a low rate when compared to those shown in literature, which vary between 9.7% and 15.8% (11, 12). This apparent discrepancy can derive from the fact that we do not provide medical care to patients on acute phases of the SCI, that we carry out a medical consultation prior to the exam and that we have excluded the patients with a greater risk of UTI due to the usage of a indwelling catheterization (14). On the other hand, even with such a low incidence, we had 21 infections among 661 patients, which makes it unlikely for a type II (low statistic power) error to have taken place on the differential analysis, even considering the total amount of investigated independent variables.

A provocative result found in the analysis of our data has to do with the level of the injury. Although we have not observed any associations between tetraplegia and the outcome of interest, this situation was changed when we dichotomized the level of the injury as T6 level, or as above or below the T6 level. This difference can derive from the fact that the detrusor sphincter dyssynergy and the autonomic dysreflexia are more frequent on injuries above the thoracic sympathetic chain. In fact, we have found, both in univariate and multivariate analyses, three times more chance of occurrence of UTI after the UDS in patients with an injury that is T6 or higher, in relation to those with an injury that is T7 or lower. It is also interesting to highlight that the extent of SCI, here evaluated through the ASIA Impairment Scale, has not influenced this result, as already described on a previous study (15).

It is known that patients with tetraplegia require greater daily care, which often involves hiring caregivers, increasing the general risk of UTI, although not specifically with regard to the UDS (15). A possible explanation for the higher incidence of UTI in patients with a T6 level injury or higher could reside on a bladder ischemia that would occur in two situations: increase in the intravesical pressure (low complacency and / or high detrusor pressure) or when there is vesical hyperdistension (16). Patients with a T6 level injury or higher present detrusor sphincter dyssynergy, which enhances the resistance to urine drainage, keeping the detrusor pressure high for a longer period of time, which would diminish even more the vesical wall's perfusion. This alteration in the blood flow would retard the liberation of leukocytes and other agents of antibacterial defense interfering with the protection barriers against a bacterial invasion (translocation) and/or bacteremia. The possibility of autonomic dysreflexia to interact with these phenomena should be considered. A study with 140 patients has shown by logistic regression a slight association between autonomic dysreflexia and UTI after UDS in patients with SCI (15). We highlight that in our univariate and multivariate analyses the use of antimicrobials has not prevented this higher chance of UTI to occur after UDS.

Like other authors, amongst the other variables of interest we have also not identified any correlation between the outcome and the patient's age (13, 14), sex (13, 14), spasticity (14), method of bladder emptying (12, 15), asymptomatic bacteriuria (12) and time since injury (14, 15).

This study presents some limitations regarding the fact that it is based on a retrospective survey of three hospital units, geographically far from each other. On the other hand, it is a multicenter study, which has allowed us to collect an expressive number of patients, so far the largest indexed on PubMed specifically on the subject. With regard to the external validity of our results, we highlight that we have considered here only patients with traumatic SCI hospitalized in rehabilitation centers. Amongst those with a non-traumatic etiology such as, for example, spina bifida, cerebral palsy, Parkinson and others, there are possibly clinical and physiopathological differences of the neurogenic bladder that might lead to a daring extrapolation of these results.

Based on a retrospective hospital cohort of patients with traumatic SCI we have demonstrated that the use of prophylactic antimicrobials would not lower the risk of UTI as a complication of UDS. The only independent variable that could be associated with that outcome was the level of the injury: T6 or above. Even in these patients the use of antimicrobials would not be justified.
